# Association of *CHI3L1* gene variants with YKL‐40 levels and hypertension incidence: A population‐based nested case‐control study in China

**DOI:** 10.1111/jcmm.16148

**Published:** 2020-12-06

**Authors:** Tian Xu, Xiaowei Zheng, Aili Wang, Zhirong Guo, Yonghong Zhang

**Affiliations:** ^1^ Department of Neurology Affiliated Hospital of Nantong University Nantong China; ^2^ Department of Epidemiology School of Public Health and Jiangsu Key Laboratory of Preventive and Translational Medicine for Geriatric Diseases Medical College of Soochow University Suzhou China

**Keywords:** CHI3L1 gene, hypertension, nested case‐control study, YKL‐40

## Abstract

YKL‐40 was reported to be associated with the risk of hypertension. Whether the variants of *CHI3L1* gene were associated with both YKL‐40 levels and hypertension needs to be further elucidated. In a 1:1 matched case‐control study of 507 pairs with available YKL‐40 levels and DNA samples nested in a prospective cohort of Chinese subjects, the 15 tag single nucleotide polymorphisms (SNPs) of *CHI3L1* gene were genotyped. The levels of YKL‐40 among different genotypes of each SNP were compared after false discovery rate adjustment. Multivariable conditional logistic regression analyses were used to explore the association between the genotypes and the risk of hypertension. Subjects with the genetic variants for rs10399931, rs1538372, rs2071580, rs2297839 and rs4950928 had lower YKL‐40 levels. The genetic variant for rs10399805 was associated with higher YKL‐40 level. Subjects with the genotype of GA/AA of rs10399805 had a 1.34‐fold risk of hypertension compared with those with GG genotype in the total population (*P* = .05). Subjects with heterozygote/rare homozygote genotype of rs4950928 and rs2297839 both had a significantly lower risk of hypertension compared with those with major homozygote genotype among men. The ORs (95% CIs) were 0.46 (0.23‐0.89) and 0.49 (0.26‐0.91), respectively. The above three SNPs could significantly improve the accuracy of risk prediction for hypertension based on the conventional factors. The genotypes of rs10399805, rs4950928 and rs2297839 may hopefully become stable biomarkers for predicting the risk of hypertension.

## 1 INTRODUCTION

Hypertension so far still represents a huge public health issue worldwide and it is the major risk factor for cardiovascular diseases (CVDs), including coronary heart disease, stroke and heart failure.^1^ Hypertension is a heterogeneous disease. Besides age, sex, ethnicity and other environmental factors (eg lipid levels and obesity), the genetic variation also influences this trait.^2^ For those genes encoding hypertension‐associated proteins, it is necessary for us to explore the associations between the genetic variants and the risk of hypertension because of the great significance in primary prevention.

YKL‐40, which is also known as human cartilage glycoprotein‐39 and chitinase‐like protein 1, is produced by macrophages and neutrophils within inflamed tissues. It is associated with atherosclerosis and promotes vascular smooth muscle cells attachment, spreading and migration. At present, YKL‐40 has been proposed to be a novel biomarker of inflammation, atherosclerosis and endothelial dysfunction.^3^, ^4^, ^5^ Previous cross‐sectional and prospective studies indicated that the YKL‐40 was associated with hypertension in total population and predicted the incidence of hypertension in male subjects.^6^, ^7^, ^8^ Chitinase 3‐like 1 (CHI3L1) gene, encoding YKL‐40, was reported to play a role in asthma, bronchial hyper‐responsiveness, abnormal lung function and hepatitis C virus–induced liver fibrosis,^9^, ^10^, ^11^ while no significant association between *CHI3L1* gene variants and cardiovascular diseases was observed.^5^, ^12^ In the present study, we aimed to examine the association of tag SNPs in *CHI3L1* gene with YKL‐40 levels and the incidence of hypertension in a population‐based nested case‐control study in China.

## 2 SUBJECTS AND METHODS

### 2.1 Study populations

The present study was performed as a nested case‐control study in 20343 subjects participating in a population‐based investigation of risk factors for cardiovascular diseases initiated in Changshu, Jiangsu Province of China from 2007 through 2008. Details of the study were presented elsewhere.^7^ In brief, data on demographic information, lifestyle risk factors and personal medical history were collected with standard questionnaires by the trained staff. Cigarette smoking was defined as having smoked at least 100 cigarettes before. Alcohol consumption was defined as having consumed any type of alcohol beverage at least 12 times during the last 1 year. Bodyweight and height were measured using a regularly calibrated stadiometer and balance‐beam scale with participants wearing light clothing and no shoes. Body mass index (BMI) was calculated as weight in kilograms divided by height in metres squared.

Subjects were advised to avoid alcohol drinking, cigarette smoking, coffee/tea and exercise for at least 30 minutes before their blood pressure (BP) measurements. Three BP measurements were measured by using an electronic BP monitor (Omron HEM‐770A, OMRON Healthcare Inc, Dalian, China) with a 30‐second interval. BP was measured with the subject in a sitting position after 5 minutes of rest. The mean of the three BP measurements was used in the analysis. Hypertension was defined as mean systolic BP ≥ 140 mm Hg and/or mean diastolic BP ≥ 90 mm Hg, or current use of antihypertensive medications. Individuals were excluded from the current analysis for the following reasons: baseline hypertension; prevalence or history of coronary heart disease, stroke, chronic kidney diseases, tumours, chronic obstructive pulmonary diseases or peripheral artery diseases. Finally, a total of 12423 subjects were free of hypertension, CVD or any other severe diseases at baseline.

This study was approved by the institutional review board and was conducted in accordance with the guidelines of the Declaration of Helsinki. All participants provided written informed consents.

### 2.2 Outcome definition

All participants were followed up at 2013 with an interview, examination and BP measurements. The method and equipment for three BP measurements were consistent with those at baseline. New cases of hypertension were identified in the following ways: a) self‐reported use of antihypertensive therapy in the previous two weeks with corresponding medical documentations; and b) systolic BP ≥ 140 mm Hg and/or diastolic BP ≥ 90 mm Hg during the visit. Subjects who had coronary heart diseases, stroke, chronic kidney diseases, tumours, chronic obstructive pulmonary diseases or peripheral artery diseases during the follow‐up period were excluded. Among 12423 healthy individuals at baseline, 2344 were lost to follow‐up. Finally, a total of 1774 new cases of hypertension were ascertained.

### 2.3 Genetic variant selection and genotyping

As presented before, the 700 age‐ and sex‐matched pairs were selected and their plasma YKL‐40 levels were assayed.^7^ Among them, the 507 pairs had available DNA samples for genotyping. All SNPs in the *CHI3L1* gene region and ±2kb flanking regions were screened based on the Han Chinese population of the 1000 Genomes Project. Minor allele frequency (MAF)≥0.05 was used to filter low‐frequency variants. We performed linkage disequilibrium (LD) analysis with a *r* ^2^ threshold of 0.8. Finally, 15 tag SNPs were selected in Haploview for genotyping.^13^ A multiplex PCR‐ligase detection reaction method was used for genotyping these SNPs. For each SNP, the alleles were distinguished by different fluorescent labels of allele specific oligonucleotide probe pairs. Different SNPs were further distinguished by different extended lengths at 3’end.^14^

### 2.4 Statistical analysis

Deviation of genotype distribution for each SNP from the Hardy‐Weinberg equilibrium was tested by a goodness‐of‐fit χ ^2^. *P* < .05 represented departing significantly from Hardy‐Weinberg‐expected proportions. Between matched cases and controls, baseline characteristics were compared using paired‐samples *t* test (normal distribution) or Wilcoxon signed‐rank test (skewed distribution) for continuous variables and unadjusted conditional logistic regression analyses for categorical variables, respectively.

Previous cross‐sectional studies suggested that the YKL‐40, encoded by *CHI3L1* gene, was associated with the risk of hypertension in total population.^6^, ^8^ Our prospective nested case‐control study found that YKL‐40 could be an effective biomarker for predicting the incidence of hypertension in male subjects.^7^ In the present study, the levels of YKL‐40 among subjects with different genotypes (major homozygote/heterozygote/rare homozygote) were compared by Wilcoxon signed‐rank test. The association between the genotypes, which were significantly correlated with YKL‐40 levels after false discovery rate (FDR) adjustment, and the risk of hypertension was examined under the dominant model. Multivariable conditional logistic models were used to estimate the odds ratios (ORs) and 95% confidence intervals (CIs) after adjustment for the significant variables in the univariate analyses. A multiplicative interaction term between genotypes and age (>55 or ≤55 years) or sex was set in the multivariable conditional logistic model to test the interaction effect on the risk of hypertension. The net reclassification index (NRI) and integrated discrimination improvement (IDI) were calculated to evaluate the predictive value of adding genotypes to conventional risk factors for hypertension.^15^ All statistical tests were two‐tailed and were considered significant if the *P*‐values were less than 0.05. Statistical analyses were conducted using SAS (version 9.4; SAS Institute, Cary, NC, USA) and R software (version 3.5.1; The R Foundation for Statistical Computing, Vienna, Austria).

## 3 RESULTS

All SNPs were in Hardy‐Weinberg equilibrium except rs75001356 (*P* = .04). In Table 1, new hypertension cases were more likely to be drinkers and have higher body mass index and blood pressures at baseline. As shown in Table 2, subjects with the genetic variants for rs10399931, rs1538372, rs2071580, rs2297839 and rs4950928 tended to have lower baseline YKL‐40 levels. In contrast, subjects with the genetic variant for rs10399805 were prone to have higher YKL‐40 levels at baseline.

**Table 1:** Baseline characteristics according to hypertension cases and matched controls

Variable	Cases (n = 507)	Control (n = 507)	*P*
Age (y)	51.0 ± 10.6	51.0 ± 10.6	Matched
Male, n (%)	167 (32.9)	167 (32.9)	Matched
Body mass index (kg/m^2^)	22.8 ± 3.2	21.7 ± 2.8	<.01
Systolic blood pressure (mm Hg)	123 ± 7.6	116 ± 10.5	<.01
Diastolic blood pressure (mm Hg)	77 ± 5.8	72 ± 7.3	<.01
Total cholesterol (mmol/L)	4.6 ± 1.0	4.6 ± 0.9	.72
Triglyceride (mmol/L)	1.6 ± 1.2	1.4 ± 1.0	.08
LDL cholesterol (mmol/L)	2.5 ± 0.8	2.5 ± 0.7	.93
HDL cholesterol (mmol/L)	1.3 ± 0.3	1.4 ± 0.3	.06
Fasting glucose (mmol/L)	5.1 ± 1.2	5.0 ± 1.0	.34
Smoking, n (%)	122 (24.1)	115 (22.7)`	.38
Drinking, n (%)	99 (19.5)	73 (14.4)	<.01
YKL‐40 (ng/mL)	59.0 (24.5‐126.4)	56.0 (24.7‐123.0)	.73
Glucose‐lowering therapy, n (%)	0 (0)	2 (0.39%)	.99
Lipid‐lowering therapy, n (%)	1 (0.2%)	1 (0.2%)	1.00

Note

YKL‐40 was expressed as median (interquartile range); other continuous variables were expressed as mean ± SD.

**Table 2:** Summary of associations between SNP genotypes in CHI3L1 gene and YKL‐40 levels (ng/mL) at baseline

SNP	Major homozygote/heterozygote/rare homozygote	Major homozygote^a^	Heterozygote^a^	Rare homozygote^a^	*P* ^b^	*P* ^c^
rs10399805	GG/GA/AA	46.5 (22.5‐112.9)	65.2 (28.9‐132.1)	68 (26.1‐156.8)	.0016	.0037
rs10399931	CC/CT/TT	69.1 (29.5‐129.6)	51.7 (23.6‐121.3)	33.4 (11.7‐84.2)	.0001	.0007
rs1538372	GG/GA/AA	64.4 (27.9‐127.0)	56.1 (24.2‐124.8)	37.2 (19.8‐83.8)	.0015	.0037
rs2071580	CC/CT/TT	61.4 (26.9‐126.4)	44.7 (19.3‐110.1)	31.1 (4.9‐35.0)	.0006	.0021
rs2275351	CC/CT/TT	52.9 (25.0‐118.3)	58.9 (25.1‐127.0)	60.5 (20.2‐133.3)	.74	.8633
rs2297839	CC/CT/TT	63.0 (27.7‐127.2)	45.2 (19.8‐108.4)	32.1 (5.9‐69.5)	.0005	.0021
rs2297944	AA/GA/GG	58.8 (25.0‐124.9)	47.8 (23.5‐119.7)	33.1 (31.0‐37.2)	.39	.4964
rs35405821	CC/CG/GG	55.8 (21.8‐119.7)	61.2 (26.2‐131.2)	55.3 (27.4‐113.5)	.3	.4667
rs4950928	CC/CG/GG	65.6 (28.3‐131.2)	42.5 (17.7‐83.9)	31 (3.7‐37.2)	.0001	.0007
rs77111685	CC/CA/AA	54.8 (24.0‐119.7)	64.1 (27.9‐140)	101.2 (29.0‐166.2)	.11	.2200
rs883125	CC/CG/GG	53.5 (24.7‐122.2)	59.7 (23.6‐128)	67.2 (33.7‐132.2)	.39	.4964
rs903359	GG/GT/TT	58.6 (24.7‐123.6)	51.4 (25.4‐124.7)	68.9 (21.6‐120.4)	.93	.9300
rs946260	AA/GA/GG	59.7 (24.7‐125.3)	52.2 (25.0‐123.6)	61.6 (21.6‐120.4)	.83	.8938
rs946261	CC/CT/TT	55.7 (21.2‐124.7)	60.7 (26.5‐126.3)	69.1 (30.6‐113.5)	.27	.4667

^a^ Median(interquartile range) of YKL‐40 levels among genotypes;

^b^ Wilcoxon signed‐rank test; and

^c^ *P‐*values of false discovery rate (FDR).

**Figure 1:**
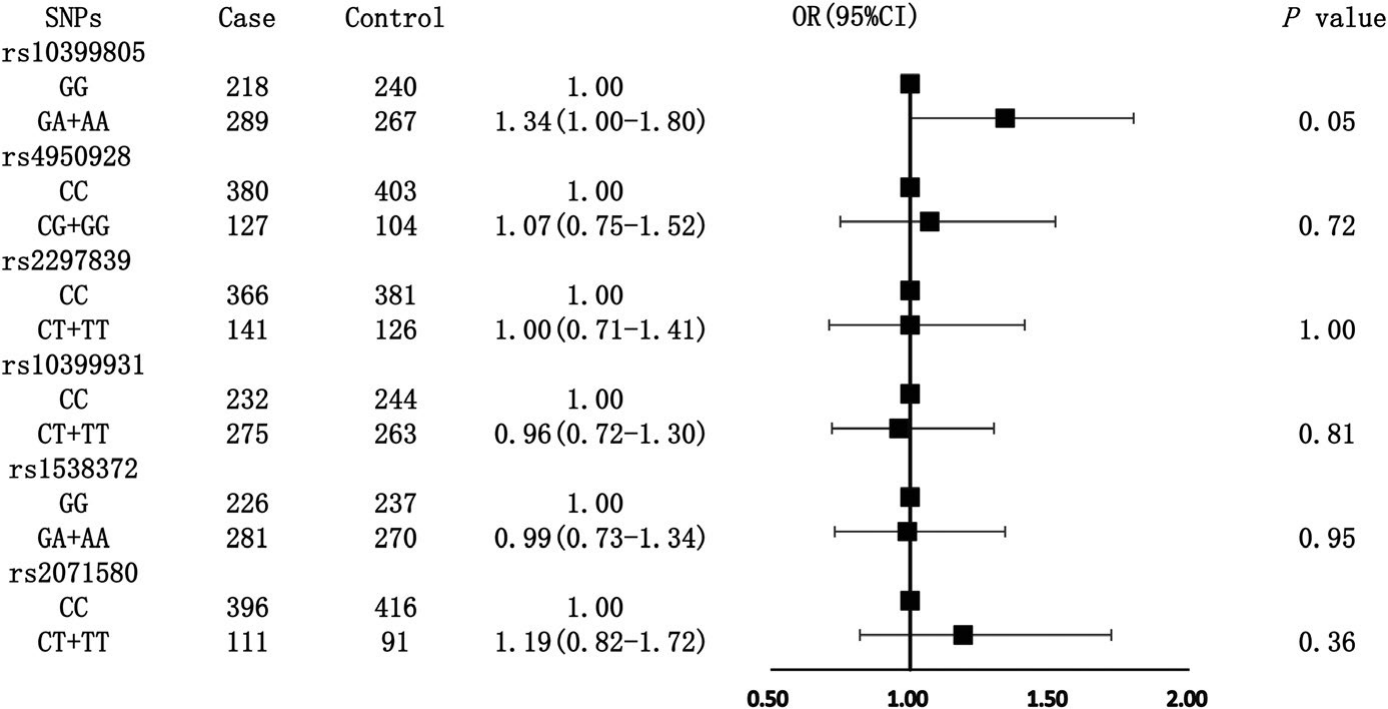
Associations between six SNPs and the risk of hypertension among total population. The adjusted confounding factors included body mass index, blood pressure and drinking

**Figure 2:**
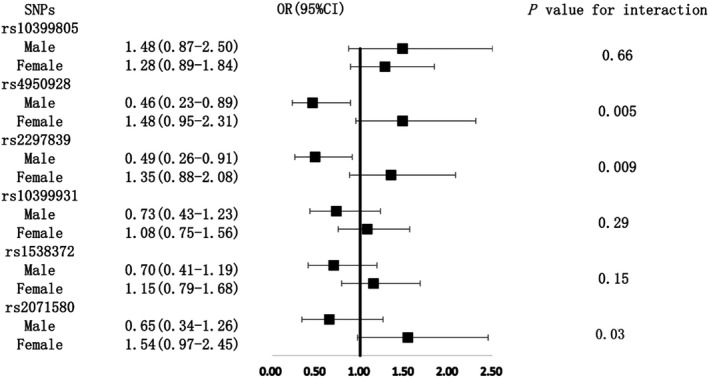
Associations between six SNPs and the risk of hypertension among male and female subgroups. The adjusted confounding factors included body mass index, blood pressure and drinking

**Figure 3:**
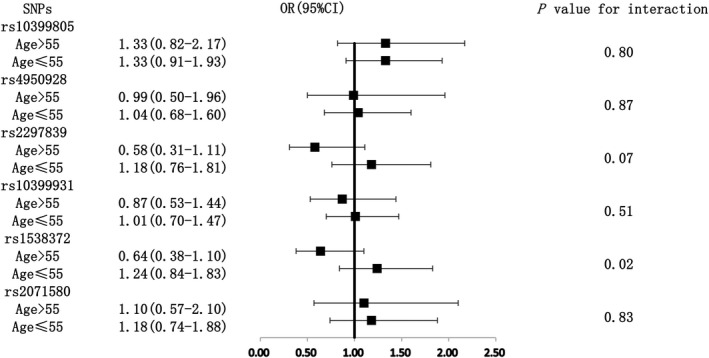
Associations between six SNPs and the risk of hypertension among age >55 and ≤55 subgroups. The adjusted confounding factors included body mass index, blood pressure and drinking

Among the above SNP genotypes significantly associated with YKL‐40 levels after the FDR adjustment, subjects with the genotype of GA/AA of rs10399805 had a 1.34‐fold risk of hypertension compared with the GG genotype subjects in the total population on the boundary of significance (*P* = .05) (Figure 1). Subgroup analyses indicated that subjects with heterozygote/rare homozygote genotype of rs4950928 and rs2297839 both had a significantly lower risk of hypertension compared with those with major homozygote genotype among men. The ORs (95% CIs) were 0.46 (0.23‐0.89) and 0.49 (0.26‐0.91), respectively. A significant interaction was also observed between rs4950928, rs2297839 and sex (Figure 2), while the other three SNP (rs10399931, rs1538372 and rs2071580) genotypes were not associated with the risk of hypertension in total population and different sex or age subgroups (Figures 1, 2, 3).

As shown in Table 3, adding the rs10399805 genotype to a conditional logistic model consisting of conventional risk factors significantly improved the accuracy of risk prediction for hypertension in the total population (NRI = 58.78%, *P* < .01; IDI = 0.33%, *P* = .027). Similar predictive effect was also observed when adding the rs2297839 (NRI = 65.87%, *P* < .01, IDI = 1.53%, *P* = .005) or rs4950928 (NRI = 68.26%, *P* < .01, IDI = 1.59%, *P* = .004) genotype to conventional risk factors for predicting hypertension among men.

**Table 3:** The comparison regarding prediction performance between multivariate‐adjusted models with and without taking three SNP genotypes into consideration

	NRI (%)	IDI (%)
rs10399805 (total population)		
Conventional factors^a^	1.00 (reference)	1.00 (reference)
Conventional factors + rs10399805	58.78 (47.01‐70.54)	0.33 (0.04‐0.63)
*P*	<.01	.027
rs2297839 (Male population)		
Conventional factors^a^	1.00 (reference)	1.00 (reference)
Conventional factors + rs2297839	65.87 (45.62‐86.12)	1.53 (0.47‐2.60)
*P*	<.01	.005
rs4950928 (Male population)		
Conventional factors^a^	1.00 (reference)	1.00 (reference)
Conventional factors + rs4950928	68.26 (48.1‐88.42)	1.59 (0.49‐2.68)
*P*	<.01	.004

Abbreviations: IDI, integrated discrimination improvement; NRI, net reclassification index.

^a^ Conventional factors included body mass index, baseline blood pressures and drinking.

## 4 DISCUSSION

Our prospective nested case‐control study indicated that the six SNP genotypes of *CHI3L1* gene were associated with YKL‐40 levels. We firstly reported that the subjects with heterozygote/rare homozygote genotype of rs10399805 had a marginally higher risk of hypertension in the total population. Those with heterozygote/rare homozygote genotype of rs2297839 and rs4950928 were at a significantly lower risk of hypertension among men.

Among the six SNPs significantly associated YKL‐40 levels in the present study, four SNPs (rs10399805, rs10399931, rs1538372 and rs4950928) located within the promoter or 5’ untranslated region and the other two (rs2071580 and rs2297839) were in the intron. A few of previous studies have focused on the association between the *CHI3L1* gene variants and YKL‐40 levels and yielded the similar results with ours. Kaspar et al^16^ analysed eight *CHI3L1* SNP genotypes and YKL‐40 levels in rheumatoid arthritis and healthy individuals. Four SNPs (rs10399805, rs10399931, rs1538372 and rs4950928) were duplicated with our study. The significant associations between these four SNP genotypes and YKL‐40 levels were consistent in two studies. In another study among Chinese population, two SNPs (rs10399931 and rs10399805) were genotyped and also correlated with YKL‐40 levels.^17^ According to the web‐based SNP analysis tools of SNPinfo (http://snpinfo.niehs.nih.gov/) ^18^ and DNase‐seq and RegulomeDB (http://www.regulomedb.org/),^19^ we speculated that the above six SNPs may be functional due to the potential transcriptor binding sites and DNase I peaks.^20^

*CHI3L1* gene was reported to play an important role in asthma, bronchial hyper‐responsiveness, abnormal lung function and hepatitis C virus–induced liver fibrosis.^9^, ^10^, ^11^ While the researches focusing on the association between *CHI3L1* gene and CVD were limited and yielded the negative results, a prospective study investigated 41 SNPs of *CHI3L1* gene and CVD outcomes including all major vascular events, CVD death, all‐cause mortality, myocardial infarction and thromboembolic stroke. However, no significant association was observed.^5^ Another study^12^ in Chinese population also reported that *CHI3L1* rs4950928 was not associated with peripheral artery disease risk and its long‐term mortality. Our present study is the first one to detect the association between the *CHI3L1* SNPs and the risk of hypertension based on a nested case‐control study with a median follow‐up of five years, which considerably reduces potential biases inherent in cross‐sectional or retrospective studies. Furthermore, the significant associations of rs4950928 and rs2297839 with hypertension among men were consistent with our previous study focusing on YKL‐40 levels and hypertension.^7^ Considering the variability of YKL‐40 levels among the subjects, the genotypes of rs10399805, rs4950928 and rs2297839 may hopefully become more stable biomarkers for predicting hypertension.

Some limitations of this study should also be mentioned. First, it is difficult for us to estimate the accurate onset time of hypertension because all participants were followed up only one time. Second, we presume that the marginal association between rs10399805 and the risk of hypertension in total population is attributed to the limited sample size of the present study. Third, it is unavoidable that there is selection bias because of the nested case‐control design. Large sample prospective cohort studies were necessary to validate the stability and robustness of our findings across different genetic models in the future. Furthermore, due to the number of subgroup analyses performed, statistical significance may have occurred by chance alone.

In conclusion, six *CHI3L1* SNPs (rs10399931, rs1538372, rs2071580, rs2297839, rs4950928 and rs10399805) were associated with YKL‐40 levels. Among total population, subjects with the genetic variant of rs10399805 had a marginally higher risk of hypertension. Subjects with the genetic variants of rs4950928 and rs2297839 were at a significantly lower risk of hypertension among men. The above three *CHI3L1* SNPs were helpful to predict the risk of hypertension.

## CONFLICT OF INTEREST

The authors confirm that there are no conflicts of interest.

## AUTHOR CONTRIBUTIONS

**Tian Xu:** Conceptualization (equal); Data curation (equal); Funding acquisition (equal); Investigation (equal); Methodology (equal); Project administration (equal); Supervision (equal); Validation (equal); Writing‐original draft (equal); Writing‐review & editing (equal). **Xiaowei Zheng:** Data curation (equal). **Aili Wang:** Data curation (equal). **Zhirong Guo:** Data curation (equal). **Yonghong Zhang:** Conceptualization (equal).

## Data Availability

The data are free access to available upon request.
